# Predictors of Access to Early Support in Families of Children with Suspected or Diagnosed Developmental Disabilities in the United Kingdom

**DOI:** 10.1007/s10803-023-05996-7

**Published:** 2023-05-04

**Authors:** Suzi J. Sapiets, Richard P. Hastings, Vasiliki Totsika

**Affiliations:** 1 Centre for Educational Development, Appraisal and Research (CEDAR), University of Warwick, Coventry, CV4 7AL UK; 2 Tizard Centre, University of Kent, Canterbury, Kent, CT2 7NZ UK; 3 Department of Psychiatry, Monash Medical Centre, Monash University, Block P, Level 3 246 Clayton Rd, Clayton, VIC 3168 Australia; 4 Division of Psychiatry, University College London, Maple House, 149 Tottenham Court Rd, London, W1T 7BN UK

**Keywords:** Early intervention, Early identification, Service provision, Intellectual disability, Autism, Special educational needs

## Abstract

This study examined predictors of access to early support amongst families of 0-6-year-old children with suspected or diagnosed developmental disabilities in the United Kingdom. Using survey data from 673 families, multiple regression models were fitted for three outcomes: intervention access, access to early support sources, and unmet need for early support sources. Developmental disability diagnosis and caregiver educational level were associated with intervention access and early support access. Early support access was also associated with child physical health, adaptive skills, caregiver ethnicity, informal support, and statutory statement of special educational needs. Unmet need for early support was associated with economic deprivation, the number of household caregivers, and informal support. Multiple factors influence access to early support. Key implications include enhancing processes for formal identification of need, addressing socioeconomic disparities (e.g., reducing inequalities, increasing funding for services), and providing more accessible services (e.g., coordinating support across services, flexible service provision).

Early support is the provision of support to ensure optimal child development during early childhood (0–6 years), including specific interventions and programmes to improve child and family outcomes, and contact with various support services across education, health, social care, voluntary, community and other service sectors (Akhmetzyanova, 2016; Dunst, 2007; Harbin et al., 2000; McWilliam, 2016). The provision of early support for children with developmental disabilities (e.g., developmental delay, intellectual disability, autism) and their families can improve a range of child and family outcomes (Fuller & Kaiser, 2020; Skotarczak & Lee, 2015). However, research evidence suggests there are low levels of access to early support amongst children with developmental disabilities and their families (e.g., Birkin et al., 2008; Bromley et al., 2004; Grant & Isakson, 2013; McManus et al., 2014, 2020; Rosenberg et al., 2008; Ruble et al., 2005; Vohra et al., 2014; Yingling & Bell, 2020).

To address disparities of access, it is important to identify what factors predict different aspects of access to early support (access to interventions, access to support services, and unmet need for support services, which we broadly refer to as access to early support). Unmet need for support has been defined as ‘myriad situations in which children and their families are unable to access needed health [and other] services for the child (e.g., prescription medication, therapy services) or the family (e.g., respite care, family mental healthcare)’ (Lindly et al., 2016, p.713). Unmet need is typically measured by parental caregiver report of support needed but not accessed. While parents might underreport unmet need (Magnusson et al., 2016), in general this measure is the most straightforward, cost-effective way to obtain insight into unmet need, especially when conducting research with families that are not in contact with support services.

Existing research suggests several factors influence access to early support for families of children with developmental disabilities, such as caregiver ethnicity, educational attainment, and economic resources, primary language spoken at home, and child age, gender, ethnicity, and individual needs (Kasilingam et al., 2019; Khetani et al., 2017; McIntyre & Zemantic, 2017; McManus et al., 2014, 2019; Marshall et al., 2016; Nygren et al., 2012; Roberts et al., 2008; Rosenberg et al., 2008; Sapiets et al., 2021). Furthermore, unmet need for early support is predicted by families’ access to services, elements of family-centered healthcare, caregiver educational attainment, and child age, ethnicity, and health needs (Kasilingam et al., 2019; Magnusson & Mistry 2017; Magnusson et al., 2016).

Current research on predictors of access to early support is limited both in number and scope of studies. First, different measurements of early support and unmet need are used across studies, which are often related to specific interventions or therapies (Kasilingam et al., 2019; McIntyre & Zemantic 2017; Magnusson & Mistry, 2017; Magnusson et al., 2016; Nygren et al., 2012) rather than provision across various support systems (i.e., health, education, social care, and other service sectors). Further, broad or dichotomous measurements are often used, such as receipt of an individualized family support or education plan, receipt of Part C services, caregiver report of qualification for the use of special therapies, or unmet need for specific therapies or interventions (Magnusson & Mistry, 2017; Magnusson et al., 2016; McManus et al., 2014; Marshall et al., 2016; Roberts et al., 2008; Rosenberg et al., 2008), which may not account for variation in access to or unmet need for early support. Whilst the findings of these studies are informative, there is a lack of research capturing access to a range of early support provisions across the various service systems.

Second, the samples of several studies consist only of families whose children have already received a developmental disability diagnosis (e.g., Kasilingam et al., 2019; McIntyre & Zemantic, 2017; Nygren et al., 2012; Sapiets et al., 2021). In these studies, it is not possible to explore whether receipt of a diagnosis impacts access to early support. Furthermore, the findings of these studies do not account for factors impacting access to support in families whose children have not yet received a diagnosis. Considering one of the main aims of early support is prevention, such as early identification of developmental disabilities and reduction of the risk of secondary health and psychosocial difficulties experienced by children and families (e.g., Royal Australasian College of Physicians, 2013), it is important to investigate access among everyone, not just those with established diagnoses. Last, no studies have been conducted in the United Kingdom (UK). As the set-up of service systems varies across countries, inevitably impacting access to support (Sapiets et al., 2021), UK-based research evidence is needed.

The prevalence of developmental disabilities in the UK may impact access to early support. Approximately 2.5% and 1.9–3.2% of children in the UK have an intellectual disability or autism, respectively (McConkey, 2020; Office for National Statistics, 2020). Amongst children in UK schools, there is considerable co-occurrence of intellectual disability and autism and the number of autism diagnoses has increased over the past decade (Kinnear et al., 2019; McConkey, 2020; Roman-Urrestarazu et al., 2021).

The structure of services in the UK may also impact access to early support. A range of service systems (health, education, social care, voluntary, community, etc.) and support approaches (e.g., Denne et al., 2018) are involved in early support for children with developmental disabilities in the UK. In this context, health and education systems play key roles in early support, mainly through universally free services designed to serve the UK population. Contact with (and referrals from) primary services are often required to access specialist services in the UK, including services that assess developmental disabilities and support a range of child and family needs. Research suggests the complexity of the support system and fragmented approach support across services, limited capacity and availability of services, regional differences in service provision, a postal/zip code lottery, and delays to assessment and diagnostic processes are barriers of access to early support in the UK (e.g., Chadwick et al., 2002; Crane et al., 2016; Howlin & Moore, 1997; Karim et al., 2012; Ridding & Williams, 2019; Sapiets et al., 2021; Sapiets et al., 2023).

Government policies and guidelines advocating for early support may also impact access, for example healthy child programs (Department of Health and Social Care, 2009), education reforms (Children and Families Act, 2014; Department for Education, 2012; Special Educational Needs and Disability Code of Practice, 2014), and professional guidelines from the National Institute for Health and Care Excellence (2011, 2013, 2016, 2018). A key aspect of the education reforms was for timely assessment of special educational needs (SEN) and the integration of support into a single education, health, and care plan. These statutory plans (also referred to as statutory statements of SEN) are formal recognition of the child’s SEN and document the child’s legal entitlements to support across education, health, and social care. While these policies aim to improve access to early support, difficulties obtaining a statutory plan, government funding cuts to services and the implementation of austerity appear to impact access to support (Cullen & Lindsay, 2019; Karim et al., 2012; Sapiets et al., 2021; Sapiets et al., 2023).

In the present study, we examined predictors of access to early support in families of young children with suspected or diagnosed developmental disabilities across the UK. This addresses limitations of previous research by utilizing three comprehensive measurements of access to early support, such as access to interventions, access to various support sources (professionals and services across education, health, social care, and other support services in the early years) and unmet need for various support sources (support wanted but not accessed). In addition, we focus explicitly on the early years, include families of children with suspected developmental disabilities and those not presently receiving support, and provide UK specific evidence to add to the international literature.

## Methods

We designed a survey to collect cross-sectional data on families’ access to early support in the previous 12 months in addition to a range of child, family, and service factors. To ensure the supports measured in the survey was comprehensive and the terminology reflected service provision across the four UK nations at the time of data collection, the survey was developed with input from a group of parental caregivers of children with developmental disabilities in addition to a range of professionals and a third-sector organizations supporting families of children with developmental disabilities. Ethical approval was granted by the University of Warwick’s Humanities and Social Sciences Research Ethics Committee (reference 57/17-18). Participants were recruited via social media and distribution via several organizations working with families in the UK. Recruitment took place between September 2018 and May 2019.

### Participants

Overall, 673 parental caregivers of children with suspected or diagnosed developmental disabilities aged 0–6 years completed the survey anonymously (see Table 1 for participant characteristics). Overall, 83.4% (*n* = 561) of the children had received a developmental disability diagnosis and 16.6% (*n* = 112) had not. The most common developmental disabilities children had received a diagnosis of (or were suspected to have) were autism, intellectual disability, developmental delay, and/or social communication disorder. For full details of participants with diagnosed or suspected developmental disability diagnoses or labels see Table 2 in Sapiets et al. (2023).

**Table 1:** Participant characteristics

Participant characteristics (*N* = 673)	Total N (%) *or* Mean (SD)
*Child factors*	
Child age (years)	4.8 (1.5) range 0.1–6.9
Child sex [male]	481 (71.5)
Child health conditions	1.4 (1.3) range 0–5
Child adaptive skills (GO4KIDDS total score)	21.4 (7.6) range 8–39
*Family factors*	
Caregiver ethnicity group [ethnic minority group]	98 (14.6)
Caregiver disability [no disability]	410 (60.9)
Caregivers in the household [1 caregiver]	132 (19.6)
Caregivers’ educational level [≥ 1 caregiver educated to degree level or higher]	338 (50.2)
Family economic deprivation	1.5 (1.1) range 0–4
Caregivers’ employment^a^ [≥ 1 caregiver in employment]	543 (80.7)
Income poverty^a^ [> poverty line]	195 (29.0)
Subjective poverty^a^ [not managing financially]	105 (15.6)
Ability to raise money^a^ [would struggle to raise money]	405 (60.2)
Other disabled children in the family [no other disabled children]	477 (70.9)
Informal support sources	3.6 (2.4) range 0–12
Perceived helpfulness of informal support	3.7 (0.8) range 1.3-5.0
*Service factors*	
Developmental disability diagnosis [no diagnosis]	112 (16.6)
Statutory statement of SEN [no statement]	332 (49.3)

*Notes.* SEN = special educational needs. ^a^Variable used as an indicator of family economic deprivation

Child factors included: age (years), sex (male/female), adaptive skills (GO4KIDDS total score), and health conditions (count of up to five physical health conditions: visual impairment, hearing impairment, epileptic seizures, mobility problems, other). Family factors included: caregiver ethnicity (ethnic minority group/White ethnic majority group, i.e., White British/English/Welsh/Scottish/Northern Irish), caregiver disability (yes/no), caregivers in household (one/two caregivers), caregivers’ educational level (≥ 1/<1 caregiver in household educated to degree level or higher), family economic deprivation (count of four indicators of economic deprivation: caregiver unemployment, income poverty, subjective poverty, and financial hardship; with higher scores indicating increased economic deprivation), other disabled children in household (yes/no), informal support sources (count of up to 12 informal support sources), and perceived helpfulness of informal support (mean helpfulness rating of informal support sources, with higher scores indicating increased helpfulness). Service factors included developmental disability diagnosis (diagnosis/no diagnosis), and statutory statement (statement/no statement)

### Materials

#### Predictor Variables

Overall, 14 child, family, and service factors were included as predictor variables (see Table 1). Child factors included: age (years), sex (male/female), adaptive skills (GO4KIDDS total score; Perry et al., 2015), and number of physical health conditions (count of up to 5 physical health conditions: visual impairment, hearing impairment, epileptic seizures, mobility problems, other). Family factors included: caregiver ethnicity (ethnic minority group/White ethnic majority group, i.e., White British/English/Welsh/Scottish/Northern Irish), caregiver disability (disabled yes/no, measured in accordance with UK disability definitions; Government Statistical Service Harmonisation Team, 2019), number of caregivers in household (one/two caregivers), caregivers’ education level (at least one caregiver in household educated to degree level or higher/no caregiver in household educated to degree level), family economic deprivation (economic deprivation composite score, see below), other disabled children in household (yes/no), informal support sources (count of up to 12 informal support sources in the preceding 12 months, e.g., partner, friends, other parents, based on the Family Support Scale; Dunst, 1984), and perceived helpfulness of informal support (mean helpfulness rating of informal support sources, with higher scores indicating increased helpfulness, derived from the Family Support Scale; Dunst, 1984). Service factors included: developmental disability diagnosis (at least one diagnosed developmental disability/no diagnosed developmental disability), and statutory statement (receipt of statutory statement of SEN/no statutory statement of SEN), both as indicators of services’ formal identification of child need.

Four indicators of family economic deprivation were used, caregiver unemployment, income poverty, subjective poverty, and financial hardship. To ascertain caregivers’ employment, participants were asked to indicate their (and their partner’s, if living with a partner) work status and a dichotomous variable was created to indicate family caregiver employment (at least one caregiver in employment/no caregiver in employment). Family income was equivalized using the modified Organization for Economic Cooperation and Development scale (n.d.), which adjusts a household’s income based on the number of adults and children in the household. Income poverty was defined as households with an equivalized income below 60% the UK median equivalized income (£28,400 at the time of data collection; Office for National Statistics, 2019). This was used to create a dichotomous variable for income poverty (equivalized income above poverty the line/equivalized income below the poverty line).

Subjective poverty (managing financially/not managing financially) was measured by asking participants to indicate their current financial management as: (1) living comfortably, (2) doing alright, (3) just about getting by, (4) finding it quite difficult, or (5) finding it very difficult. A variable was created to dichotomize responses 1–3 as ‘managing financially’ and 4–5 as ‘struggling financially’. Financial hardship (family could raise £2,000 in an emergency/family would struggle to raise money) was measured by asking participants about their perceived ability to raise £2,000 for a hypothetical emergency as: (1) I could easily raise the money, (2) I could raise the money, but it would involve some sacrifices (e.g., reduced spending, selling a possession), (3) I would have to do something drastic to raise the money (e.g., selling an important possession), or (4) I don’t think I could raise the money. A variable was created to dichotomize responses 1–2 as ‘could raise the money’ and 3–4 as ‘would struggle to raise the money’. These four dichotomous variables (caregiver employment, income poverty, subjective poverty, financial hardship) were combined to provide a count of indicators of family economic deprivation (0–4), with higher scores indicating higher economic deprivation. Participants with missing data on two or more of these indicators were not included in the combined variable (*n* = 11, 1.6%). Overall, family economic deprivation scores ranged from 0 to 4 (*M* = 1.5, *SD* = 1.1), indicating variation in participants experiences of the four indicators of family economic deprivation (see Table 1 for further details).

#### Outcome Variables

Three outcome variables were included: intervention access (intervention access/no intervention access), access to early support sources (count of access to up to 49 early support sources), and unmet need for early support sources (count of unmet need for up to 27 key support sources; Sapiets et al., 2023).

For intervention access, participants were asked to list any interventions they or their child had received in the preceding 12 months, either to support their child’s development or to support them as parental caregivers. A few interventions were listed as examples to help participants complete the question (e.g., Early Bird, Hanen®, Incredible Years®, Triple P^TM^, Applied Behavior Analysis, SCERTS®, TEACCH®, therapy, counseling). Participants’ free-text responses were coded against a pre-specified definition of intervention as a packaged intervention or multi-sessional support program (Sapiets et al., 2023), unless explicitly covered in the measure of early support sources described below. We created a dichotomous variable which identified if the participant had or had not accessed an intervention based on our definition.

To measure access to early support sources, a comprehensive list of 49 early support sources was included in the survey, presented in three groups: (a) 27 key professionals across education, health, and social care (e.g., school staff, general medical practitioner, occupational therapist, speech and language therapist, social worker, respite carer), (b) 10 additional health specialists (e.g., neurologist, ophthalmologist, podiatrist), and (c) 12 other supports (e.g., parent groups, telephone helplines, children’s centers; Sapiets et al., 2023). Participants were asked to indicate if they had accessed any of these support sources in the preceding 12 months. We created a variable to count the number of early support sources the family had accessed from this list (possible range 0–49).

If a participant reported they *had not* accessed support from any one of the 27 key professionals in the past 12 months, they were asked if they had wanted support from the professional they had not accessed. Unmet need was defined as support wanted but not accessed (i.e., if the participant reported they had not accessed support from the professional **and** wanted support from the professional). We created a variable to count unmet need for early support sources the participant wanted but had not accessed (possible range 0–27; Sapiets et al., 2023).

### Procedure

Multiple regression models were fitted for the three outcome variables: binary logistic regression for intervention access (binary variable), multiple linear regression for access to early support sources (count variable distributed fairly normally), and negative binomial regression for unmet need for early support (count variable). Participants with missing data on any of the predictor or outcome variables were excluded from analyses. Overall, 566 participants were included in the analysis for intervention access and 567 participants were included in the analyses for access to early support sources and unmet need for early support.

## Results

Table 2 reports the descriptive statistics for the outcome variables. The majority of participants had not accessed an intervention (*n* = 545, 81.0%). The mean number of early support sources accessed by participants was 14.6 (*SD* = 5.7, range 0–32). The mean number of support sources reported as an unmet need was 3.2 (*SD* = 3.2, range 0–17).

**Table 2:** Participants’ access to support

Outcome variables (*N* = 673)	Total N (%) *or* Mean (SD)
Intervention access	127 (18.9) intervention access
Access to early support sources	14.6 (5.7) range 0–32
Unmet need for early support sources	3.2 (3.2) range 0–17

*Notes.* Three outcome variables were included: intervention access (intervention access/no intervention access), access to early support sources (count of access to up to 49 early support sources), and unmet need for early support sources (count of unmet need for up to 27 key support sources)

### Intervention Access

Table 3 reports the results of the binary logistic regression model for intervention access. Receipt of a developmental disability diagnosis and caregivers’ educational level were significant independent predictors of intervention access. Families of children with a developmental disability diagnosis were more likely than those without a diagnosis to access an intervention (*b* = 1.027, *OR* = 2.792, *p* = .013). Families without a caregiver educated to at least degree level were less likely than those with a caregiver educated to at least degree level to access an intervention (*b* = -0.617, *OR* = 0.539, *p* = .008).

**Table 3:** Binary logistic regression model of intervention access

				95% CI for *OR*	
Predictor variables [reference group]	*B*	Sig.	*OR*	Lower	Upper
*Child factors*					
Child age	0. 057	. 594	1.059	0. 858	1. 307
Child sex [male]	0. 119	0.629	1.127	0.695	1.827
Child adaptive skills	-0.015	0.401	0.985	0.952	1.020
Child health conditions	-0.113	0.224	0.893	0.744	1.072
*Family factors*					
Caregiver ethnicity group [ethnic minority group]	0.198	0.520	1.219	0.667	2.231
Caregiver disability [no disability]	0.160	0.492	1.174	0.743	1.855
Caregivers in household [1 caregiver]	0.398	0.214	1.489	0.795	2.790
Caregivers’ educational level [≥ 1 caregiver educated to degree level or higher]	-0.617	0.008*	0.539	0.341	0.853
Family economic deprivation	0.127	0.260	1.135	0.910	1.416
Other disabled children [no other disabled children]	0.176	0.482	1.192	0.730	1.947
Informal support sources	0.031	0.546	1.031	0.933	1.139
Perceived helpfulness of informal support	-0.238	0.078	0.788	0.605	1.027
*Service factors*					
Developmental disability diagnosis [no diagnosis]	1.027	0.013*	2.792	1.247	6.250
Statutory statement [no statement]	0.089	0.722	1.093	0.668	1.789

*Notes.* CI = Confidence Interval. OR = Odds Ratio. * p = < 0.05

Fourteen predictor variables were included in the model of intervention access (intervention access/no intervention access). Child factors included: age (years), sex (male/female), adaptive skills (GO4KIDDS total score), and health conditions (count of up to five physical health conditions: visual impairment, hearing impairment, epileptic seizures, mobility problems, other). Family factors included: caregiver ethnicity (ethnic minority group/White ethnic majority group, i.e., White British/English/Welsh/Scottish/Northern Irish), caregiver disability (yes/no), caregivers in household (one/two caregivers), caregivers’ educational level (≥ 1/<1 caregiver in household educated to degree level or higher), family economic deprivation (count of four indicators of economic deprivation: caregiver unemployment, income poverty, subjective poverty, and financial hardship; with higher scores indicating increased economic deprivation), other disabled children in household (yes/no), informal support sources (count of up to 12 informal support sources), and perceived helpfulness of informal support (mean helpfulness rating of informal support sources, with higher scores indicating increased helpfulness). Service factors included developmental disability diagnosis (diagnosis/no diagnosis), and statutory statement (statement/no statement)

### Access to Early Support Sources

Table 4 reports the results of the multiple linear regression model for access to early support sources. Significant independent predictors of access to early support sources were receipt of a developmental disability diagnosis, receipt of a statutory statement, child health conditions, child adaptive skills, caregiver ethnicity group, caregivers’ educational level, and informal support sources. Increased access to early support sources was found for families of children with a developmental disability diagnosis compared to those without a diagnosis (*b* = 1.306, β = 0.084, *p* = .019), with a statutory statement compared to those without a statement (*b* = 2.469, β = 0.218, *p* < .001), lower child adaptive skills (*b* = -0.110, β = -0.150, *p* < .001), a higher number of child physical health conditions (*b* = 1.780, β = 0.400, *p* < .001), and a higher number of informal support sources (*b* = 0.428, β = 0.170, *p* < .001). Decreased access to early support sources was found for families with a primary caregiver from an ethnic minority group compared to a White ethnic majority group (*b* = -1.275, β = -0.081, *p* = .016) and families without a caregiver educated to at least degree level compared to those with one or more caregiver educated to at least degree level (*b* = -0.812, β = -0.072, *p* = .048).

**Table 4:** Multiple linear regression model of access to early support sources

				95% CI for β	
Predictor variables [reference group]	*B*	Sig.	β	Lower	Upper
*Child factors*					
Child age	-0.118	0.525	-0.027	-0.484	0.247
Child sex [male]	0.802	0.064	0.062	-0.048	1.652
Child adaptive skills	-0.110	< 0.001**	-0.150	-0.171	-0.050
Child health conditions	1.780	< 0.001**	0.400	1.467	2.092
*Family factors*					
Caregiver ethnicity group [ethnic minority group]	-1.275	0.016*	-0.081	-2.314	-0.235
Caregiver disability [no disability]	0.290	0.474	0.025	-0.506	1.087
Caregivers in household [1 caregiver]	-0.335	0.512	-0.023	-1.339	0.669
Caregivers’ educational level [≥ 1 caregiver educated to degree level or higher]	-0.812	0.048*	-0.072	-1.617	-0.007
Family economic deprivation	-0.121	0.515	-0.025	-0.487	0.244
Other disabled children [no other disabled children]	0.576	0.190	0.046	-0.287	1.439
Informal support sources	0.428	< 0.001**	0.170	0.247	0.610
Perceived helpfulness of informal support	-0.010	0.965	-0.002	-0.472	0.451
*Service factors*					
Developmental disability diagnosis [no diagnosis]	1.306	0.019*	0.084	0.214	2.397
Statutory statement [no statement]	2.469	< 0.001**	0.218	1.601	3.336

*Notes.* CI = Confidence Interval. * p = < 0.05 ** p < .001

Fourteen predictor variables were included in the model of access to early support sources (count of access to up to 49 early support sources). Child factors included: age (years), sex (male/female), adaptive skills (GO4KIDDS total score), and health conditions (count of up to five physical health conditions: visual impairment, hearing impairment, epileptic seizures, mobility problems, other). Family factors included: caregiver ethnicity (ethnic minority group/White ethnic majority group, i.e., White British/English/Welsh/Scottish/Northern Irish), caregiver disability (yes/no), caregivers in household (one/two caregivers), caregivers’ educational level (≥ 1/<1 caregiver in household educated to degree level or higher), family economic deprivation (count of four indicators of economic deprivation: caregiver unemployment, income poverty, subjective poverty, and financial hardship; with higher scores indicating increased economic deprivation), other disabled children in household (yes/no), informal support sources (count of up to 12 informal support sources), and perceived helpfulness of informal support (mean helpfulness rating of informal support sources, with higher scores indicating increased helpfulness). Service factors included developmental disability diagnosis (diagnosis/no diagnosis), and statutory statement (statement/no statement)

### Unmet Need for Early Support

Table 5 reports the results of the negative binomial regression model for unmet need for early support. Significant independent predictors of unmet need for early support were the number of caregivers in the household, family economic deprivation, informal support sources, and helpfulness of informal support. Increased unmet need for early support was found in families with one rather than two caregivers in the household (*b* = -0.366, *RR* = 0.693, *p* = .007), higher family economic deprivation (*b* = 0.101, *RR* = 1.107, *p* = .033), fewer informal support sources (*b* = -0.084, *RR* = 0.920, *p* = .001), and lower helpfulness of informal support (*b* = -0.140, *RR* = 0.870, *p* = .023).

**Table 5:** Negative binominal regression model of unmet need for early support sources

				95% CI for *RR*	
Predictor variables [reference group]	*B*	Sig.	*RR*	Lower	Upper
*Child factors*					
Child age	0.089	0.067	1.094	0.994	1.204
Child sex [male]	0.158	0.173	1.171	0.933	1.468
Child adaptive skills	-0.014	0.090	0.986	0.971	1.002
Child health conditions	-0.004	0.919	0.996	0.916	1.082
*Family factors*					
Caregiver ethnicity group [ethnic minority group]	0.039	0.783	1.039	0.789	1.369
Caregiver disability [no disability]	-0.038	0.723	0.963	0.780	1.188
Caregivers in household [1 caregiver]	-0.366	0.007*	0.693	0.531	0.906
Caregivers’ educational level [≥ 1 caregiver educated to degree level or higher]	0.140	0.188	1.151	0.934	1.418
Family economic deprivation	0.101	0.033*	1.107	1.008	1.215
Other disabled children [no other disabled children]	-0.219	0.051	0.804	0.645	1.001
Informal support sources	-0.084	0.001**	0.920	0.877	0.965
Perceived helpfulness of informal support	-0.140	0.023*	0.870	0.771	0.981
*Service factors*					
Developmental disability diagnosis [no diagnosis]	0.104	0.465	1.109	0.840	1.464
Statutory statement [no statement]	0.142	0.216	1.152	0.921	1.442

*Notes.* CI = Confidence Interval. RR = Rate Ratio. Dispersion parameter = 1 (negative binomial dispersion parameter set by SPSS). * p = < 0.05 ** p < .001

Fourteen predictor variables were included in the model of unmet need for early support sources (count of unmet need for up to 27 key support sources). Child factors included: age (years), sex (male/female), adaptive skills (GO4KIDDS total score), and health conditions (count of up to five physical health conditions: visual impairment, hearing impairment, epileptic seizures, mobility problems, other). Family factors included: caregiver ethnicity (ethnic minority group/White ethnic majority group, i.e., White British/English/Welsh/Scottish/Northern Irish), caregiver disability (yes/no), caregivers in household (one/two caregivers), caregivers’ educational level (≥ 1/<1 caregiver in household educated to degree level or higher), family economic deprivation (count of four indicators of economic deprivation: caregiver unemployment, income poverty, subjective poverty, and financial hardship; with higher scores indicating increased economic deprivation), other disabled children in household (yes/no), informal support sources (count of up to 12 informal support sources), and perceived helpfulness of informal support (mean helpfulness rating of informal support sources, with higher scores indicating increased helpfulness). Service factors included developmental disability diagnosis (diagnosis/no diagnosis), and statutory statement (statement/no statement)

## Discussion

We examined predictors of access to early support and unmet need for early support among a comparatively large sample of families of young children with suspected or diagnosed developmental disabilities in the UK. A crucial finding is that formal identification of child disability (receipt of developmental disability diagnosis and/or statutory statement) predicted access to early support sources, as did lower levels of adaptive skills and a higher number of physical health conditions. This indicates access to early support is partly based on child level of need (e.g., developmental, health) and services’ formal identification of child need, which is consistent with previous literature (Khetani et al., 2017; McIntyre & Zemantic, 2017; McManus et al., 2014, 2019; Marshall et al., 2016; Sapiets et al., 2021). This finding extends the existing evidence base as most previous studies only included children who had already received a formal diagnosis. It is promising that statutory recognition of need (i.e., statutory statement receipt) predicted access to early support, indicating related UK legislation may contribute to promoting access to support (e.g., Children and Families Act, 2014). Of these variables, the only factor that predicted intervention access was developmental disability diagnosis, and none of these variables predicted unmet need for early support. The lack of relationship between these factors and unmet need indicates formal identification of child disability and child level of need may not be associated with caregivers’ perceptions of unmet need for early support across key education, health, and social care professionals.

Caregiver education also predicted both access to intervention and early support sources. Caregivers with higher educational attainment may be more likely to access support due to an increased awareness of the need for (or potential benefit of) early support (Nygren et al., 2012). Increased educational attainment may also improve caregivers’ abilities to navigate service systems and advocate for early support. Mixed findings on caregiver education and access to early support have been reported in previous studies (Kasilingam et al.,^2019^; Khetani et al., 2017; McIntyre & Zemantic, 2017; Roberts et al., 2008). A recent UK study found an increased rate of autism diagnosis amongst children of mothers with increased educational attainment, therefore caregiver education may impact diagnosis receipt (Kelly et al., 2019).

Caregiver ethnicity predicted access to early support sources, but not intervention access. This indicates disparities of access based on ethnicity, which is consistent with previous studies (Khetani et al., 2017; McManus et al., 2019; Marshall et al., 2016; Nygren et al., 2012; Rosenberg et al., 2008). However, the association between ethnicity and access to support is likely more complex than the findings of this study suggest, as prior research indicates access varies according to specific ethnicity group, rather than simply minority versus non-minority status (Sapiets et al., 2021). The wider social context may also account for ethnicity disparities, such as structural racism, discrimination, and marginalization, which contribute to barriers of access experienced by people who belong to ethnic minority groups (de Leeuw et al., 2020; Čolić et al., 2021). A lack of culturally appropriate support may also account for these findings, as previous research highlights the importance of culturally appropriate support (Barnard-Brak et al., 2021; Čolić et al., 2021; Sapiets et al., 2021).

Increased informal support sources predicted access to early support sources, but not intervention access. At present there is limited research on the relationship between informal and formal early support, though this relationship appears to be related to family composition and childcare support (Chadwick et al., 2002; Chauhan et al., 2017; Sapiets et al., 2021). Increased informal support may facilitate access to early support due to practical, informational, emotional, or other support provided by informal sources. Furthermore, informal support may be directly related to formal support access (e.g., help contacting, travelling to, or paying for services) or indirectly related (e.g., helping with caregiving and other responsibilities, enabling the caregiver more time to navigate formal support systems). Perceived helpfulness of informal support did not predict access to intervention or early support sources, which suggests the quantity, rather than the quality, of informal support is associated with access to early support.

Both the number of informal support sources and the perceived helpfulness of informal support predicted unmet need for early support, with increased perceived unmet need amongst caregivers who reported fewer informal support sources and lower helpfulness of informal support. This suggests both the quantity and quality of informal support influences caregivers’ perceptions of unmet need for early support. Families with an increased number (and perceived helpfulness) of informal support sources may be less likely to perceive unmet need for support from sources they had not accessed, especially the social care supports (e.g., home support staff, respite care, childminder) measured.

Similarly, having one caregiver in the household (compared to two) predicted increased unmet need for early support, which might be related to increased caregiving and household responsibilities for caregivers in one-parent households, leading to caregivers perceiving (and likely needing) more formal support.

Higher levels of family economic deprivation (comprised of caregivers’ unemployment, income poverty, subjective poverty, and financial hardship) also predicted increased unmet need for early support. This highlights potential limitations of the universally-free service system in the UK. This may be due to both direct costs (e.g., the need to pay for private services because free services are not accessible) and indirect costs associated with access to support (e.g., travel, childcare).

We expected, but did not find, that family economic deprivation would predict access to both intervention and early support sources. Differences in the financial set-up of service systems likely account for this, as previous research has largely focused on contexts without a universally-free service system (Khetani et al., 2017; McIntyre & Zemantic, 2017; McManus et al., 2019; Marshall et al., 2016; Nygren et al., 2012). However, qualitative evidence has shown that families in the UK experience difficulties accessing universally-free services (Karim et al., 2012). Similar to our study, Rosenberg et al. (2008) found economic deprivation was not associated with access to early support in the United States, after controlling for developmental delay status and ethnicity, which were both associated with access. Recent research in the United States found parents of autistic children report higher unmet health care needs despite being able to access healthcare through health insurance, indicating not only the presence of higher needs, but also challenges within the healthcare system in being responsive to their needs and the family’s ability to coordinate care and identify appropriate services (Karpur et al., 2019).

### Implications

One key implication relates to the availability of formal identification for accessing support in the early years. While having a formal diagnosis is clearly important, considerable issues obtaining a diagnosis or statement have been reported (Crane et al., 2016; Cullen & Lindsay, 2019; Lamb, 2019). Therefore, it is problematic if access to early support is dependent exclusively on services’ formal identification of need. Examining and addressing barriers to formal identification of need should be a priority. For example, using telehealth to accelerate diagnostic pathways (Alfuraydan, 2021). In addition, a straightforward action to facilitate access is to provide some support whilst families await or go through formal identification processes. This may be more challenging following the Covid-19 pandemic impact on service delivery (increased pressure on services, backlog following disruptions, growing use of telehealth and remote service provision; Eapen et al., 2021; Ellison et al., 2021; Gonzales et al., 2023).

Further action is clearly needed to address disparities of access based on families’ economic status. Policies and investments to reduce poverty have the potential to reduce perceived unmet need for early support. Disability increases the risk of poverty, both broadly and for developmental disabilities (Blackburn et al., 2010; Emerson, 2004; Emerson et al., 2010), related to direct and indirect costs associated with disability (e.g., paying for specialist equipment, out-of-pocket costs associated with service access, caregivers reducing employment due to caregiving responsibilities; Cleaton et al., 2020; Dillenburger et al., 2015). Furthermore, adverse outcomes (e.g., poorer health) are associated with increased economic disadvantage (Emerson & Hatton, 2007; Totsika et al., 2021). Therefore, reducing economic disadvantage has greater implications than just for improving access to early support amongst children with developmental disabilities. It may be beneficial to explore existing policy initiatives and strategies to reduce health inequalities for people living in deprived areas of the UK, such as implementation of the Marmot review (Marmot, 2010; Marmot et al., 2020) and the A Better Start program focused on proactive community support in the early years (The National Lottery Community Fund, 2022). Key lessons include the provision of early intervention and universal services for families, involving families in the design and delivery of community services, a strong awareness of systemic racism and an anti-racist approach, and effective data and information sharing across multi-agency teams (Berry, 2023). In addition, the national roll out of evidence-based parenting programs in community settings across the UK demonstrated benefits both for families of children with and without developmental disabilities (Lindsay & Strand, 2013; Totsika et al., 2017).

Targeted interventions to improve caregivers’ knowledge and skills specific to accessing early support may also reduce disparities (cf. Cerebra, 2021; Coulman et al., 2021). However, ensuring services are more accessible is likely the most efficient action in terms of facilitating access to early support. Making services more accessible might include, for example, ensuring accessible information is available for families, accepting referrals from families in addition to services, utilizing a range of communication methods, and providing multiple supports at a single location, such as a children’s center or family hub (Anna Freud Centre, 2021; Carr & Lord, 2016; Children’s Commissioner for Wales, 2020; Sapiets, 2021). There is a clear need for more accessible services and flexible service systems that respond to the individual needs of families. This might include a professional that coordinates support across services for families, and flexibility regarding the location, timing, format, and content of provision (Dunst & Bruder, 2006; Harbin et al., 2004; Sapiets et al., 2021). Drawing existing on models of care from other contexts might be useful, such as the “medical home” in the United States (Medical Home Initiatives for Children With Special Needs Project Advisory Committee, 2002), which has demonstrated improved access to needed services and fewer unmet specialty care needs for autistic children (Farmer et al., 2014). Further research is needed to understand inequality of access based on ethnicity and other related factors (e.g., socioeconomic deprivation, perceptions of developmental disabilities). However, services can take active steps to reduce barriers and provide culturally appropriate support, such as increasing their cultural competence to reduce inadvertent discrimination, increasing professionals’ cultural skills, employing diverse and bilingual staff, and actively tackling racism (Doody & Doody, 2012; Heer et al., 2015; Magaña et al., 2021; Mir, 2010; Perepa, 2007).

### Limitations

While several factors were considered in the analyses, other factors might also influence access to early support, such as service funding and capacity, developmental surveillance processes, and professionals’ expertise (Sapiets et al., 2021). Second, as cross-sectional data were utilized, it is not possible to ascertain causal relationships between the variables examined. However, the findings provide a useful insight into outcomes of interest and multiple variables associated with them, at a specific point in time. Future studies on access to early support, especially prospective longitudinal studies, will be useful.

While our measures of access to early support addressed limitations of previous research by capturing access to a range of early support provisions across service systems, our measures did not take into account if the support accessed was appropriate, high quality, and/or met child or family needs. Furthermore, contact with multiple services is often required to obtain formal identification of developmental disabilities or other child and family needs (e.g., physical and mental health, education, social care) in the UK, therefore increased access may reflect the complexity of the early support system (e.g., disjointed approach across services, requiring referrals from primary services to access specialized professionals or services; Sapiets et al., 2023), rather than indicating improved quality of early support for families. Therefore, no assumption on the quality of support received should be made based on the findings. This should be explored in future research.

Lastly, due to convenience sampling, there is potential risk of bias. Recruitment methods may have missed some families, including those who were not in contact with services or the organizations that supported with recruitment, those not wanting support, or those unaware of (potential) child developmental disability. Therefore, our sample may be biased towards families who are in already contact with some early support sources. While the study included a diverse sample in relation to socioeconomic indicators, for example income poverty (58.4% of participants, compared to 32% of UK households in 2020; Department for Work and Pensions, 2021) and unemployment (18.4% of participants, compared to 13.4% of UK households in 2021; Office for National Statistics, 2021), there was an underrepresentation of participants from ethnic minority groups (14.6% of participants, compared to 19.4% of the population of England and Wales in 2011; Office for National Statistics, 2018). Future research could address this by designing culturally and linguistically accessible studies and targeting recruitment to promote participation from families typically underrepresented in research. Furthermore, no definition of parent or parental caregiver was provided in the study advert or information sheet, which may have impacted who took part.

## Conclusion

Multiple factors influence access to early support for families of young children with suspected or diagnosed developmental disabilities across the UK. Efforts to improve access need to be multi-pronged to reflect this, such as enhancing processes for formal identification of need, addressing socioeconomic disparities by reducing poverty and increasing funding for services, empowering families with information and practical support, and providing more accessible support across service systems.
