# Correction: Predictors of Access to Early Support in Families of Children with Suspected or Diagnosed Developmental Disabilities in the United Kingdom

**DOI:** 10.1007/s10803-024-06237-1

**Published:** 2024-02-19

**Authors:** Suzi J. Sapiets, Richard P. Hastings, Vasiliki Totsika

**Affiliations:** 1 Centre for Educational Development, Appraisal and Research (CEDAR), University of Warwick, Coventry, CV4 7AL UK; 2 Tizard Centre, University of Kent, Canterbury, Kent CT2 7NZ UK; 3 Department of Psychiatry, Monash Medical Centre, Monash University, Block P, Level 3 246 Clayton Rd, Clayton, VIC 3168 Australia; 4 Division of Psychiatry, University College London, Maple House, 149 Tottenham Court Rd, London, W1T 7BN UK

**Journal of Autism and Developmental Disorders**

10.1007/s10803-023-05996-7

In the original publication of the article, the following reference was omitted from the reference list.

Sapiets, S. J., Hastings, R. P., Stanford, C., & Totsika, V. (2023). Families’ access to early intervention and supports for children with developmental disabilities. Journal of Early Intervention, 45(2), 103–121. 10.1177/10538151221083984

This has been added in this paper.

